# Downregulation of RKIP Is Associated with Poor Outcome and Malignant Progression in Gliomas

**DOI:** 10.1371/journal.pone.0030769

**Published:** 2012-01-23

**Authors:** Olga Martinho, Sara Granja, Teresa Jaraquemada, Cláudia Caeiro, Vera Miranda-Gonçalves, Mrinalini Honavar, Paulo Costa, Margarida Damasceno, Marsha R. Rosner, José M. Lopes, Rui M. Reis

**Affiliations:** 1 Life and Health Sciences Research Institute, Health Sciences School, University of Minho, Braga, Portugal; 2 Life and Health Sciences Research Institute/3B's - PT Government Associate Laboratory, Braga/Guimarães, Portugal; 3 Department of Oncology, Hospital S. João, Porto, Portugal; 4 Department of Pathology of Hospital Pedro Hispano, Matosinhos, Portugal; 5 Institute CUF, Porto, Portugal; 6 Ben May Department for Cancer Research, University of Chicago, Chicago, Illinois, United States of America; 7 Department of Pathology, Hospital S. João, Porto, Portugal; 8 Medical Faculty of Porto University, Porto, Portugal; 9 IPATIMUP, University of Porto, Porto, Portugal; 10 Molecular Oncology Research Center, Barretos Cancer Hospital, Barretos, São Paulo, Brazil; Institute of Cancer Research, United Kingdom

## Abstract

Malignant gliomas are highly infiltrative and invasive tumors, which precludes the few treatment options available. Therefore, there is an urgent need to elucidate the molecular mechanisms underlying gliomas aggressive phenotype and poor prognosis. The Raf Kinase Inhibitory protein (RKIP), besides regulating important intracellular signaling cascades, was described to be associated with progression, metastasis and prognosis in several human neoplasms. Its role in the prognosis and tumourigenesis of gliomas remains unclear.

In the present study, we found that RKIP protein is absent in a low frequency (10%, 20/193) of glioma tumors. Nevertheless, the absence of RKIP expression was an independent prognostic marker in glioma. Additionally, by *in vitro* downregulation of RKIP, we found that RKIP inhibition induces a higher viability and migration of the cells, having no effect on cellular proliferation and angiogenesis, as assessed by *in vivo* CAM assay.

In conclusion, this is the largest series studied so far evaluating the expression levels of this important cancer suppressor protein in glioma tumors. Our results suggest that in a subset of tumors, the absence of RKIP associates with highly malignant behavior and poor survival of patients, which may be a useful biomarker for tailored treatment of glioma patients.

## Introduction

Gliomas are the most frequent primary brain tumors and include a variety of different histological tumor types and World Health Organization (WHO) malignancy grades. Histologically, astrocytic, oligodendroglial, and mixed oligoastrocytic tumors are the most relevant gliomas [1]–[3]. Low-grade (WHO grade II) diffuse astrocytomas have an invariably tendency for malignant progression to anaplastic (WHO grade III) astrocytomas and eventually to glioblastomas (WHO grade IV) – the most aggressive and frequent subtype [4]. So far, histopathology is the gold standard for the typing and grading of gliomas; however additional biological markers are needed for an advanced and more objective glioma classification, for a better prediction of prognosis and more targeted a tailored therapeutic decision-making. In this regard, to date the number of biomarkers used in neurooncology routine are rather limited to combined deletions of the chromosome arms 1p and 19q in oligodendroglial tumors, *MGMT* hypermethylation in glioblastomas and *IDH1* mutations in diffuse gliomas [5]–[7].

Raf Kinase Inhibitory Protein (RKIP; also known as PEBP1, for phosphatidylethanolamine-binding protein 1), is a widely expressed protein in normal human tissues, emphasizing its role in various physiologic processes [8], [9]. Functionally, it is an intracellular regulator of important signaling pathways such as RAF/MEK/ERK, G-protein–coupled receptor kinase-2, nuclear factor Kappa B (NFkB) and GSK3β transduction pathways [10]–[13]. Likewise, RKIP has been shown to be a multifunctional protein in carcinogenesis, being implicated in various intracellular signaling pathways that control cellular growth [14], [15], motility [16], [17], epithelial to mesenchymal transition (EMT) [18], differentiation [19], invasion and tumor metastisation [20], [21]. Initial reports have termed RKIP, as a metastasis suppressor gene, due to its paramount in the metastisation of processes of several neoplasms including melanomas and prostate [9], [22]–[24]. Further studies have shown that RKIP role is tumor-type specific, and in most cancer types, such as colorectal carcinoma, gastric adenocarcinoma, hepatocellular carcinoma, and GISTs loss of RKIP expression is linked to advanced tumor stages and worse clinical outcome [25]–[32].

With regard to RKIP expression in gliomas, two groups have reported a correlation between RKIP downregulation and higher tumor grade [28], [33]. Maresch *et al*, also suggested that RKIP expression is a marker of good prognosis in high-grade gliomas [28]. Nevertheless, the biological role of RKIP in the malignant progression of gliomas remains to be elucidated.

In the present work, using a large series of gliomas, we aimed first to clarify the frequency of RKIP expression and to validate its role in the prediction of clinical outcome in patients with glioma. Secondly, we aimed to assess, *in vitro* and *in vivo*, the biological consequences of RKIP downregulation on aggressiveness of glial tumors.

## Results

### Characterization of RKIP expression in glial tumors

**Figure 1:**
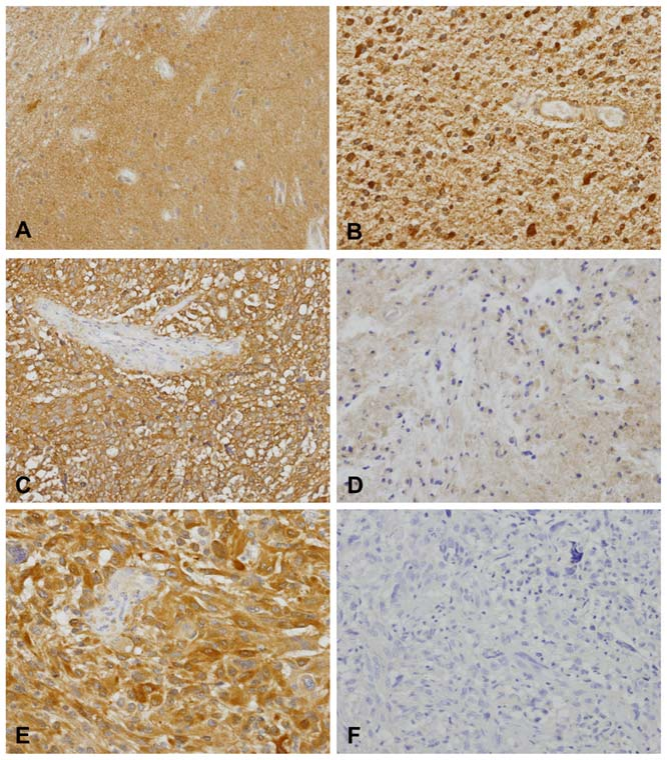
Immunohistochemistry analysis of RKIP in gliomas. **A**) Normal brain and **B**) astrocytoma grade II showing high expression. **C**) Positive and **D**) Negative expression in oligodendroglioma grade II. **E**) Positive and **F**) Negative expression in glioblastoma. All the pictures were taken with at 200× magnification.

In the present study, 193 gliomas and 18 normal brain tissues (12 were non-neoplastic brain tissues adjacent to the tumor) were studied for RKIP immunohistochemical expression. RKIP positivity was found in the cytoplasm of the great majority of samples (Figure 1), however, nuclear expression was also observed in a few cases (mainly low grade astrocytomas). We observed that RKIP was highly expressed (Figure 1A) in all the 18 non-neoplastic brain tissues studied. In general, RKIP expression was found in 89.6% (20/193) of all the tumors, specifically in 95.2% (20/21) of astrocytomas grade II (Figure 1B), in 89.5% (128/143) of glioblastomas (Figure 1E and F), in 80% (12/15) of oligodendrogliomas grade II (Figure 1C and D), 75% (3/4) of anaplastic oligoastrocytoma, and 100% of oligoastrocytomas grade II (2/2) and anaplastic oligodendrogliomas (8/8) (Table 1).

**Table 1:** RKIP expression in gliomas and associations with patients clinicopathological data (n = 193).

Parameter		RKIP Expression (N = 193)		
	N	Negative (%)	Positive (%)	*p* value
**Age (years)**				
≥50	89	10 (11.2)	79 (88.8)	0.801
<50	99	10 (10.1)	89 (89.9)	
**Gender**				
Male	117	13 (11.1)	104 (88.9)	0.672
Female	76	7 (9.2)	69 (90.8)	
**Cellular Lineage**				
Astrocytic	164	16 (9.8)	148 (90.2)	0.779
Oligodendroglial	23	3 (13)	20 (87)	
Mixed	6	1 (16.7)	5 (83.3)	
**Histological type (WHO grade)**				
Diffuse astrocytoma (II)	21	1 (4.8)	20 (95.2)	0.508
Glioblastoma (IV)	143	15 (10.5)	128 (89.5)	
Oligodendroglioma (II)	15	3 (20)	12 (80)	
Anaplastic oligodendroglioma (III)	8	0 (0)	8 (100)	
Oligoastrocytoma (II)	2	0 (0)	2 (100)	
Anaplastic oligoastrocytoma (III)	4	1 (25)	3 (75)	
**Malignancy grade (WHO)**				
Low-grade (II)	38	4 (10.5)	34 (89.5)	0.971
High-grade (III, IV)	155	16 (10.3)	139 (89.7)	
**Treatment with TMZ+RT**				
No	71	13 (18.3)	58 (81.7)	**0.004**
Yes	66	2 (3.0)	64 (97.0)	
**Follow-up (mean months** ± **SD)**				
Gliomas	181	19.2±6.2	52.0±7.9	**0.033**
Low-grade Gliomas	31	46.3±20.4	131.7±20.7	0.317
High-grade Gliomas	150	9.9±1.5	23.4±3.3	**0.025**
Glioblastomas (WHO IV)	138	10.5±1.5	16.9±1.2	0.096

N: Number of cases; SD: Standard deviation; WHO: World Health Organisation; p: person X^2^ value; TMZ: Temozolomide; RT: Radiotherapy.

**Figure 2:**
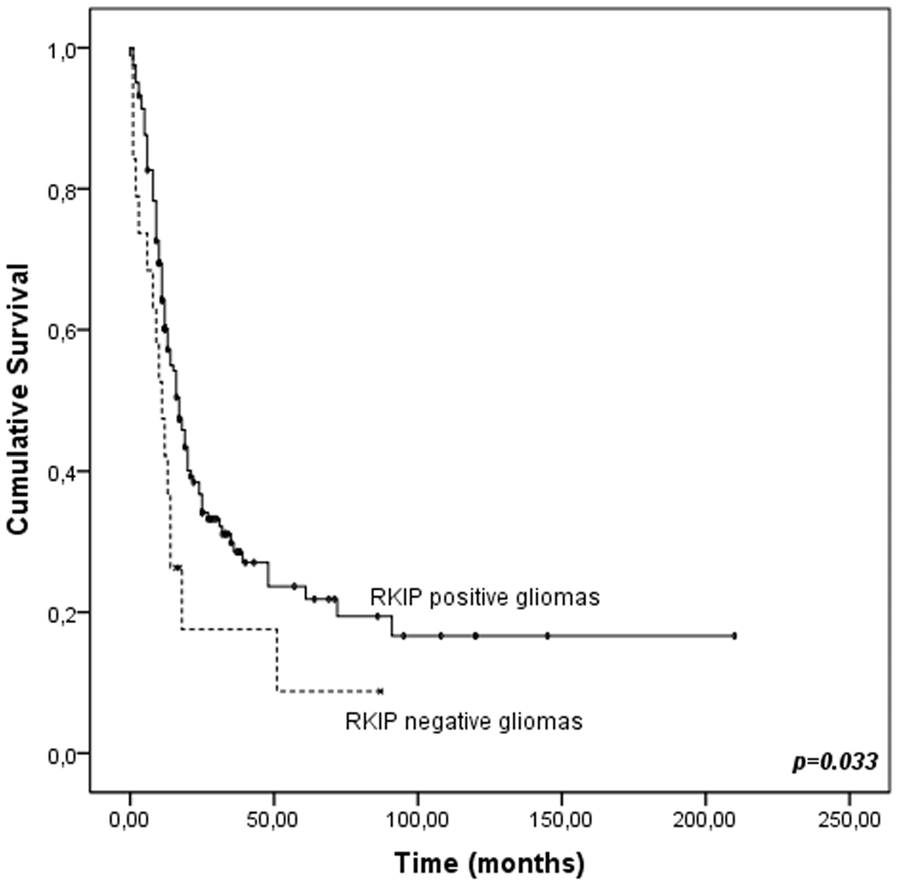
Disease-specific survival (DSS) according to RKIP expression in gliomas (n = 181). Cumulative survival is significantly lower in cases with RKIP loss of expression (p = 0.033).

No significant associations were found between RKIP expression and clinical pathological data such as age, gender, cellular lineage, histological type and malignancy grade (Table 1). However, it was found a significant (*p* = 0,004) higher number of RKIP negative cases in the subgroup of patients that were no treated with adjuvant therapy (temozolomide plus radiotherapy) (Table 1). By univariate analysis, we found a significant association (*p* = 0.033) between absence of RKIP expression and poor prognosis in gliomas (Figure 2). When the tumors were stratified by malignant grade, the association was only substantiated for the patients with high grade tumors (*p* = 0.025). When considering only the glioblastoma patients, the correlation approached significance (*p* = 0.096). Additionally, when we stratified the patients for RKIP expression and treatment simultaneously no differences were obtained between the survival of the two groups (treated *vs* no treated) of patients (p<0.05). Following multivariate analysis, we observed that the absence of RKIP expression is an independent prognostic marker for gliomas (*p* = 0.027). Additionally, age and malignancy grade were also independent prognostic markers in this cohort of glioma patients (Table 2).

**Table 2:** Independent prognostic factors in gliomas.

Parameter		Univariate Analysis		Multivariate Analysis	
	N	(months ± SD)	*p*	*HR (95% CI)*	*p*
**Malignancy grade (WHO)**					
Low-grade (II)	31	125.3±19.7		1	
High-grade (III, IV)	150	22.1±2.9	<0.001	4.9 (2.5–9.6)	<0.001
**Age (years)**					
<50	94	66.4±10.6		1	
≥50	87	16.9±1.9	<0.001	1.5 (1.01–2.2)	0.040
**RKIP expression**					
Positive	162	52.0±7.9		1	
Negative	19	19.2±6.2	0.033	1.8 (1.1–3.1)	0.027

HR: Hazard ratio, 95% CI: 95% Confidence interval.

### Effect of RKIP on glioblastoma cell biological behavior in vitro

**Figure 3:**
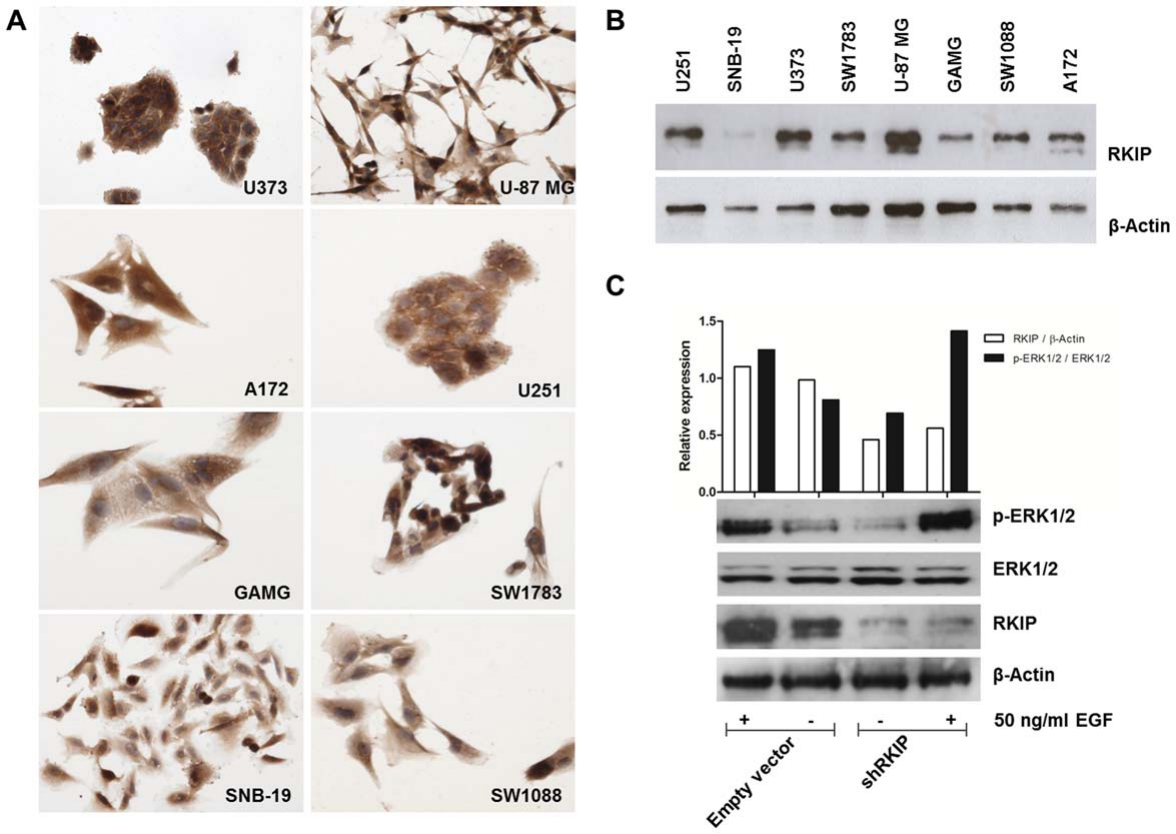
RKIP expression in glioma cell lines. **A**) Immunocytochemistry analysis of RKIP in glioma cell lines cells with both nuclear and cytoplasmic expression. **B**) Western blot analysis confirming the expression of RKIP at different levels in glioma cell lines. **C**) For RKIP inhibition, U251 cells were stably transfected with a shRNA for RKIP and with the respective empty vector for control. The band densitometry analysis showed that the shRKIP transfection induced a reduction of around 50% of the protein levels in relation to the control cells. Further, the cells were stimulated with 50 ng/ml of EGF by 10 minutes and ERK pathway activation was assessed by western blot for phospho-ERK1/2 expression. ERK pathway was overactivated in shRKIP transfected cells after EGF stimulation. Quantification of western blot results, using the band densitometry analysis, was performed with Image J software. For RKIP relative protein expression results are shown as the ratio between RKIP and β-Actin and for ERK activity the results are shown as the ratio between p-ERK1/2 and total ERK1/2.

To further explore the biological role of RKIP in glioblastoma cells, we first characterized the expression of RKIP in a panel of 8 glioblastoma cell lines. To observe the distribution of RKIP expression at the cellular level, we performed immunocytochemistry in all the glioblastoma cell lines. We observed that RKIP was present in all and the expression pattern was mainly cytoplasmatic and sometimes nuclear, mainly in mitotic cells (Figure 3A). By western blot analysis (Figure 3B), we confirm that all cell lines express RKIP, however at different levels. Next, we proceeded with the *in vitro* knockdown of RKIP using a specific short hairpin RNA (shRKIP) in the U251 cell line. As shown in Figure 3C, RKIP protein levels were downregulated in the shRKIP transfected cells in comparison with the cells transfected with the control empty vector. Since RKIP is considered to be an endogenous inhibitor of the Raf-1/MEK/ERK pathway we evaluated whether RKIP inhibition on glioblastoma cells modulates this pathway. As showed in Figure 3C, RKIP downregulated cells presented increased phosphorylation levels of ERK when stimulated with EGF, however it seems no to be significant.

**Figure 4:**
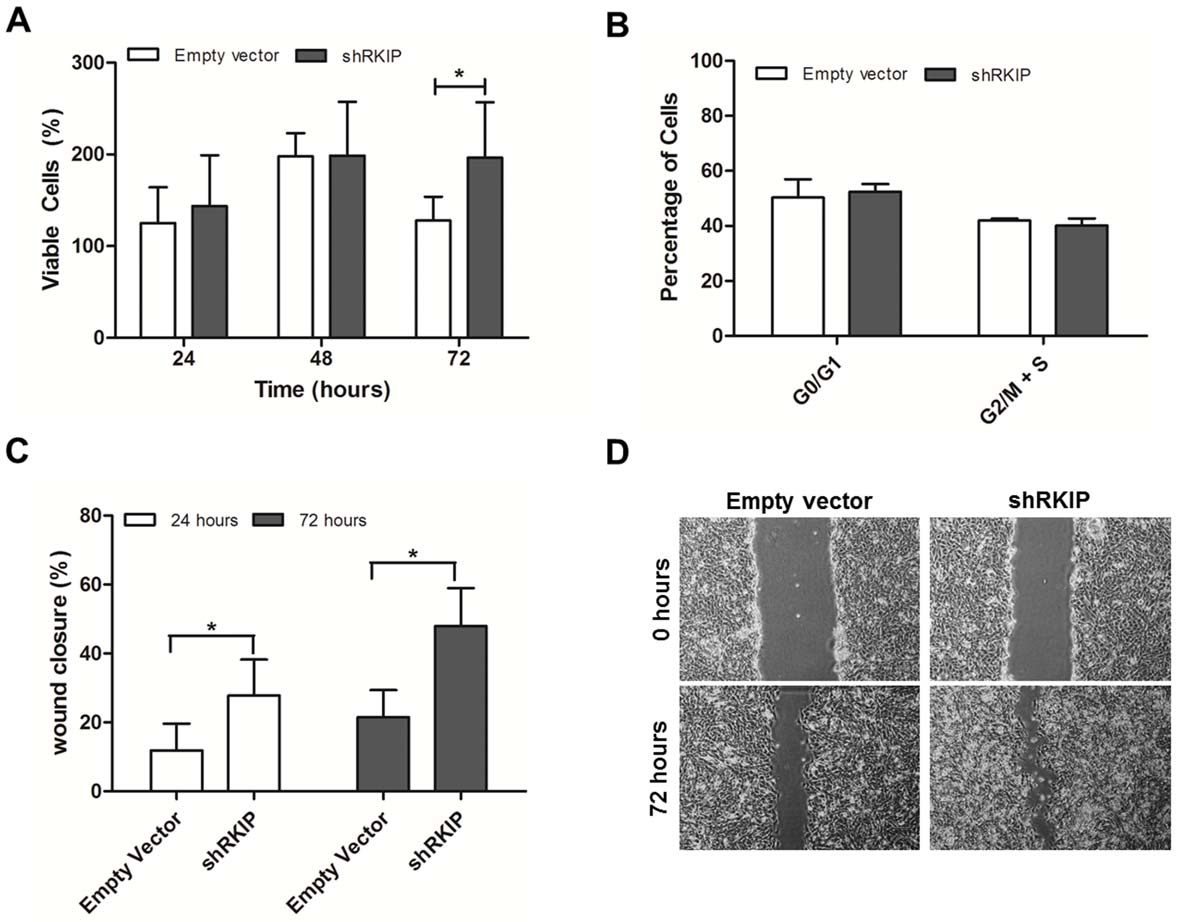
*In vitro* role of RKIP in U251 cells biological behavior. **A**) The cellular viability was measured at 24, 48 and 72 hours by MTS. RKIP inhibited cells had a viability advantage at 72 hours, when compared to control cells. **B**) Cell cycle analysis was done at 24 hour time point by flow cytometric analysis of propridium iodide stained cells. No differences were found in the cell cycle distribution. **C**) In the wound healing migration assay, a standardized scratch (wound) was applied to monolayers and digital images were taken at several time points (0, 24 and 72 hours). We observed that shRKIP transfected cells had a migration advantage at 24 and 72 hours. **D**) Representative images of the assay at 0 and 72 hours are represented (40× magnification). All the experiments were done in triplicate at least three times. Data is represented as the mean ± SD and differences with a *p*<0.05 on the Student's t test were considered statistically significant (*).

Concerning the biological assays, we first evaluated the effect of RKIP inhibition on glioblastoma cells viability over the time (Figure 4A). We found that at 72 hours the empty vector cells loose viability, while RKIP inhibited cells remained viable with a statistically significant difference (p<0.05). To evaluate whether RKIP modulates rate of cell cycle transit, we determined the cell cycle scattering of the transfected cells. As observed in Figure 4B, no statistically significant differences were found in the cell cycle scattering of shRKIP and empty vector cells.

In order to study the effect of RKIP on glioblastoma cellular migration we performed a wound healing assay (Figure 4D). We found that RKIP downregulated cells migrate significantly (*p*<0.05) more than control cells, both at 24 and 72 hours (Figure 4C).

### In vivo role of RKIP expression in glioblastomas

**Figure 5:**
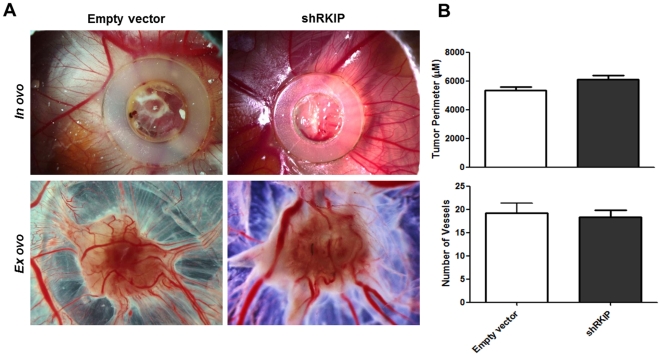
*In vivo* role of RKIP in U251 cells growth and angiogenesis. **A**) Representative pictures (16× magnification) of CAM assay after 7 days of tumor growth *in ovo* and *ex ovo*. **B**) Tumor growth was measured *in vivo* by CAM assay as described in materials and methods section. We observed a larger perimeter (µM) in the tumors formed by U251 shRKIP cells, there was no significant difference from control cells (upper panel). The counting of the blood vessels *ex ovo* revealed no differences in the number of vessels recruited in the tumors formed by shRKIP cells when compared to the control (lower panel). We analyzed 18 eggs (7 were injected with empty vector and 11 with shRKIP U251 transfected cells). The data is represented as the mean ± SD and differences with a *p*<0.05 on the Student's t test were considered statistically significant.

To evaluate the effect of RKIP-mediated tumor growth and angiogenesis *in vivo*, we performed a CAM assay. The U251 transfected cells were implanted into the CAM of the chick embryo (empty vector cells, n = 7; shRKIP cells, n = 11), and seven days after cell implantation, the chicken embryos were sacrificed to evaluate tumor growth and angiogenesis *ex ovo* (Figure 5A). The mean perimeter of the tumors formed by the control and the shRKIP U251 transfected cells was 5820.5±1265.3 µm and 6108.5±1005.7 µm, respectively, with no statistically significant differences (Figure 5B, upper panel).

To evaluate the impact of RKIP on angiogenesis, we counted *ex ovo* the number of vessels around the tumors. The mean of 19±5 and 18±4 vessels in the tumors formed by the empty vector and the shRKIP transfected cells, respectively (Figure 5B, lower panel), was not statistically significant.

## Discussion

Diffusely infiltrating gliomas are one of the most devastating cancers because they often show locally aggressive behavior and cannot be cured by existing therapies [4]. Like cancer in general, gliomas develop as a result of genetic alterations that accumulate throughout tumor progression [1]–[3]. Therefore, the elucidation of these molecular mechanism, in particular the one associated with cellular migration and invasion are crucial for a better prediction of glioma patients outcome and response to therapies [34].

RKIP protein is an important regulator of tumor cell invasion and metastasis [9], [22]–[24]. Furthermore, was reported to be a prognostic biomarker for a number of tumors including prostate, colorectal, GISTs, gastric adenocarcinoma of the intestinal subtype, hepatocellular carcinoma, pancreatic ductal adenocarcinoma and also in high grade gliomas [25]–[32]. Thus, in the present work, our goal was to evaluate the prognostic value of RKIP in glioma patients' and assessed its biological impact on gliomagenesis by *in vitro* and *in vivo* assays.

By immunohistochemistry analysis in 193 glioma tumors, we found that RKIP protein was highly expressed in all the non-neoplastic brain samples, and also in the great majority of glioma tumors with 89.6% (173/193) of positively stained cases. No significant correlations were found when the cases were stratified by age, gender, cellular lineage, histological type and malignancy grade. Our results are partially divergent with previous reports that described a correlation between loss of RKIP expression and higher malignant grade [28], [33]. By proteomic analysis in 15 cases (5 astrocytomas grade II, 5 grade III and 5 grade IV), Gimenez and colleagues described RKIP as one of the proteins that is downregulated in high grade when compared to low-grade tumors [33]. However the low number of cases may account for the discrepancy with our study. Maresch *et al*, in a recent publication using 159 gliomas, found that RKIP is present in 82% (22/27) of low-grade astrocytomas and only in ∼53% (67/126)of high grade gliomas, and the difference was statistically significant [28]. Despite using the same antibody for the immunohistochemistry analysis, the authors used a different set of criteria for scoring results. They counted a case as positive only when 90% of the cells showed moderate to strong cytoplasmic staining [28], which may outcome in false negative cases and account for the discrepancy with our data. This discrepancy was observed mainly for high grade gliomas that are heterogeneous tumors for which the immunohistochemistry classification has to take into account both extent and intensity of the staining. Noteworthy, the criteria used in our study, was the same as those used in the major reports on RKIP immunohistochemistry expression that should be used for comparison studies [21], [31], [35]. Nevertheless, Maresch *et al* found an association between loss of RKIP expression and poor prognosis of high-grade glioma patients, consistent with our findings in the present work. Moreover, in our work we propose RKIP as an independent prognostic marker for gliomas.

RKIP is widely expressed in normal human tissues and has been studied for several years as an important regulator of several physiologic processes [9]. In the central nervous system, RKIP is present in several regions of the brain and its downregulation is associated with deregulation of neurological homeostasis, being mainly implicated in Alzheimer disease [8]. Its specific function in brain tumors development is unknown.

To understand the biological role of RKIP downregulation in glioblastomas, we performed an *in vitro* and *in vivo* study with the U251 cell line transfected with a specific shRNA for RKIP. We observed *in vitro* that RKIP inhibited cells had a viability advantage at 72 hours when compared with cells with normal levels of RKIP. However, the cell cycle analysis did not disclose significant differences on the cell cycle distribution between shRKIP and empty vector cells, and in *vivo*, it did not also disclosed differences in the proliferation rates of the tumors produced by both transfected cells. Those results suggested that RKIP has no effect on glioblastoma proliferation, being the effect that we are seeing in the MTS assay a reflection of an increased metabolic activity of the cells or a decrease on cellular apoptosis. RKIP expression is negatively regulated by SNAIL [36], thus permitting enhanced NF-kB signaling resulting in a circuitry that regulates both the metastatic cascade and resistance to apoptosis by cytotoxic drugs [37]. Thus, loss of RKIP expression in cancers can result in a dramatic inhibition of apoptosis and the development of chemoresistance [38]–[40]. The role of RKIP in glioma cells apoptosis at basal conditions, without a cytotoxic stress, has to be elucidated in the future.

Additionally, we found *in vitro* that downregulation of RKIP significantly increased cellular migration, but no differences were observed in the *in vivo* vascularization of the tumors formed by both shRKIP and empty vector cells. These results suggest that RKIP can be an important promoter of glioblastoma cells migration, but has no effect in tumoral angiogenesis.

Our present findings are in accordance with previous reports in other types of tumors, where RKIP seems to be more important in migration of the cells, instead of as a proliferation suppressor [16], [21], [41]–[43]. Concerning angiogenesis, our results are distinct from reports on breast and prostate cancer mouse models, where RKIP overexpression is described as decreasing angiogenesis and vascular invasion [20], [21].

In conclusion, we herein substantiate, in the largest series studied so for, that loss of RKIP expression is an independent marker of poor clinical outcome in glioma patients. Despite the small number of RKIP negative cases, we found, by *in vitro* and *in vivo* evaluation, that RKIP inhibition is mainly associated with higher migration of glioblastoma cells. Altogether, our results suggest that in addition to its prognosis value, RKIP can be considered a modulator of the malignant phenotype in glioblastomas.

## Materials and Methods

### Tissue samples

Representative formalin-fixed paraffin-embedded blocks from 193 consecutive neurosurgeries performed due to glioma were retrieved from pathology archives of the Department of Pathology of Hospital S. João, Porto and Hospital Pedro Hispano, Matosinhos. The tumors were classified according to the WHO criteria [1]. This cohort includes 164 astrocytic, 23 oligodendroglial and 6 oligoastrocytic tumors of diverse malignant grades (Table 1). The mean age of patients at diagnosis was 50±15 (range, 2–77 years), with a female/male ratio of 0.65. Follow-up data was available in 181 patients (range: 0–210 months, mean: 22.1±28.0 months). For 137 glioblastomas we had available information regarding patients' treatment with adjuvant therapy (temozolomide plus radiotherapy) (Table 1).

### Cell lines and cell culture procedures

In the present study we used 8 glioblastoma cell lines. The cell lines SW1088, SW1783, U87-MG and A172 were obtained from ATCC (American Type Culture Collection), the cell lines SNB-19 and GAMG were obtained from DSMZ (German Collection of Microorganisms and Cell Cultures) and the cell lines U251 and U373 were kindly provided by Professor Joseph Costello, California University, Neurosurgery Department, San Francisco, USA. All cell lines were maintained in Dulbecco's Modified Eagle's Medium (DMEM 1×, High Glucose; Gibco, Invitrogen) supplemented with 10% Fetal Bovine Serum (FBS; Gibco, Invitrogen) and 1% penicillin/streptomycin solution (Gibco, Invitrogen), at 37°C and 5% CO_2_.

### Immunohistochemistry and immunocytochemistry analysis of RKIP

Representative 3 µm-thick tissue sections were used to immunohistochemical analysis according to the streptavidin-biotin peroxidase complex system (UltraVision Large Volume Detection System Anti-Polyvalent, HRP; LabVision Corporation), as previously described [32], [44]. Briefly, deparaffinised and rehydrated slides were submitted to heat-induced antigen retrieval for 20 minutes at 98°C with 10 mM citrate buffer (pH 6.0). After incubation with the primary antibody raised against RKIP (dilution 1∶600 incubation 1H at RT; Upstate Biotechnology), the secondary biotinylated goat anti-polyvalent antibody was applied for 10 minutes followed by incubation with the streptavidin-peroxidase complex. The immune reaction was visualized by 3,3′-Diamonobenzidine (DAB) as a chromogen. All sections were counterstained with Gill-2 haematoxylin. For negative controls, primary antibodies were omitted and also replaced by a universal negative control antibody (CEA, rabbit anti-human, DAKO Corporation). A prostate carcinoma was used as positive control.

Sections were scored double-blind (by JML and OM) for cytoplasmic expression following a semi-quantitative criterion, by comparison with (internal/external) positive and negative controls included in each run. The score used was the sum of the percentage of positive cells (0, negative; 1, less than 25% positive cells; 2, 26% to 50% positive cells; and 3, more than 50% positive cells) and the staining intensity (0, negative; 1, weak; 2, moderate; 3, strong). Scores between 0 and 2 were classified as negative, 3 and 4 as moderate positive, and 5 and 6 as strongly positive [32], [44]. Controversial cases were re-evaluated and classified by consensus.

For RKIP immunocytochemical analysis of glioma cell lines, the cells were plated on glass coverslips placed into 12-well plates, and allowed to adhere overnight. Then, the cells were fixed in paraformaldehyde at 4% for 15 minutes, followed by permeabilization with 0.05% Triton X-100 for 4 minutes at room temperature. The immunocytochemistry procedure was performed using a streptavidin-biotin peroxidase complex method as described above.

### Generation of a shRKIP stably expressing cell line

For generation of a glioma cell line stably expressing shRKIP, we used the PQY15 vector, containing a 19 bp shRNA for RKIP, as previously described [45], [46]. The transfection was done using the FUGENE HD reagent (Roche) as recommended by the manufacture, with 2 µg of plasmid at a ratio of 6∶2 (Reagent∶Plasmid). The cells (1.5×10^5^) were plated onto a 12-well plate until 80% confluence and transfected in DMEM medium, without FBS or antibiotics addition, for 24 hours. Then, the stable transfectants were selected with 1 µg/ml of puromycin in complete DMEM medium. The empty vector was also transfected as control.

### Western blot analysis

The cells were plated in a 6 well plate at a density of 5×10^5^ cells per well and allowed to adhere at least 24 hours. The cells were serum starved for 6 hours before protein isolation. When necessary the cells were also stimulated with 50 ng/ml of EGF for 10 minutes before the end of the 6 hours of starvation. Cells were scraped in cold PBS and lysed in buffer containing 50 mM Tris pH 7.6–8, 150 mM NaCl, 5 mM EDTA, 1 mM Na3VO4, 10 mM NaF, 10 mM NaPyrophosphate, 1% NP-40 and 1/7 of Protease cocktail inhibitors (Roche). Western blotting was done using standard 12% SDS-PAGE gel, loading 20 µg of protein per lane, with detection by enhanced chemiluminescence (SuperSignal West Femto Maximum Sensivity Substrate, Pierce). RKIP expression was evaluated using a specific antibody against RKIP (dilution 1∶2000, Upstate Biotechnology). Activated ERK was assessed using the antibody phospho-p44/42 MAPK (Thr202/Tyr204) (dilution 1∶1000, Cell Signaling Technology). The total form of ERK was also assessed with the antibody p44/42 MAPK (Erk1/2) (137F5) (dilution 1∶1000, Cell Signaling Technology). For a loading control we used β-Actin (dilution 1∶300, Santa Cruz Biotechnology). All the primary antibodies were incubated overnight at 4°C. Quantification of western blot results using the band densitometry analysis was performed with Image J software.

### Cell viability assay

The cells were plated into 96-well plates in triplicate at a density of 1×10^3^ cells per well and allowed to adhere overnight in complete DMEM medium. After 6 hours of serum starvation the viable cells were quantified using Cell Titer96 Aqueous cell proliferation assay (Promega), and used as time 0 of the experience. Then, cells were incubated in DMEM medium without serum for 24, 48 and 72 hours and cell viability was again assessed by Cell Titer96 Aqueous cell proliferation assay. The results were calibrated to the starting viability (time 0 h, considered as 100% of viability) and expressed as the mean ± SD. The assay was performed in triplicate at least three times.

### Wound healing migration assay

The cells were seeded in 6-well plates and cultured to at least 95% of confluence. Monolayer cells were washed with PBS and scraped with a plastic 200 µL pipette tip and then incubated with fresh DMEM medium without FBS. The “wound” areas were photographed by phase contrast microscopy at 0, 24 and 72 hours' time points. The relative migration distance was calculated by the following formula: percentage of wound closure (%) = 100 (A–B)/A, where A is the width of cell wounds before incubation (0H), and B is the width of cell wounds after incubation. Results are expressed as the mean ± SD. The assay was done in triplicate at least three times.

### Cell cycle analysis

The cells were plated in a 6-well plate at a density of 2×10^5^ cells per well and allowed to adhere overnight. After 6 hours of serum starvation the cells were incubated with fresh DMEM medium without serum during 24 hours. Cells were trypsinized and fixed in 70% ethanol for at least 30 minutes and then stained for 1 hour at 50°C with propidium iodide (PI) solution (20 µg/mL of PI and 250 µg/mL of RNAse in a solution of 0.1% Triton X-100 in PBS). Cell cycle analysis of the PI stained cells was performed by flow cytometry (LSRII, BD Biosciences). The percentage of cells in each phase of the cell cycle was determined with the software FlowJo version 7.6.3. The results were expressed as the mean ± SD of the percentage of cells in G1 phase or G2/M plus S phase. The assay was done in triplicate at least three times.

### Chick chorioallantoic membrane (CAM) assay

To assess *in vivo* tumor proliferation and angiogenesis we used the CAM assay as previously described [47], with some brief modifications. Fertilized chicken eggs were incubated at 37°C and 70% humidity, and on day 3 of development, a window was made into the shell, which was sealed with tape, and the eggs were returned to the incubator. On day 9 of development, small plastic rings were placed on the CAM and on day 10 of development 3×10^6^ cells, ressuspended in 20 µl of DMEM medium, were injected in the rings over the CAM. On day 17 of development, the tumor formed was photographed *in ovo* using a stereomicroscope (Olympus S2x16). The chickens were sacrificed at −80°C for 10 minutes, and the CAM and tumors were fixed with paraformaldehyde at 4% and photographed *ex ovo*. The perimeter of the tumors was measured using Cell B software (Olympus), and blood vessels were manually counted.

### Statistical analysis

Correlations between RKIP expression and clinical data of the patients were performed using the chi-square test (χ2-test). Cumulative survival probabilities were calculated using the Kaplan-Meier method. Differences between survival rates were tested using the log-rank test. Multivariate analysis was done using the Cox proportional hazards model. The statistical analysis was performed using SPSS software for Windows, version 17.0.

For *in vitro* assays, single comparisons between the different conditions studied were done using Student's t test. Statistical analysis was done using Graph Pad Prism version 5. The level of significance in all the statistical analysis was set at *p*<0.05.
